# Phosphorylation of Eukaryotic Initiation Factor 4G1 (eIF4G1) at Ser1147 Is Specific for eIF4G1 Bound to eIF4E in Delayed Neuronal Death after Ischemia

**DOI:** 10.3390/ijms23031830

**Published:** 2022-02-06

**Authors:** Emma Martínez-Alonso, Natalia Guerra-Pérez, Alejandro Escobar-Peso, Lorena Peracho, Rocío Vera-Lechuga, Antonio Cruz-Culebras, Jaime Masjuan, Alberto Alcázar

**Affiliations:** 1 Department of Research, Hospital Universitario Ramón y Cajal, IRYCIS, Ctra. Colmenar km 9.1, 28034 Madrid, Spain; natalgue@ucm.es (N.G.-P.); alejandro.escobar@hrc.es (A.E.-P.); lorena.peracho@salud.madrid.org (L.P.); 2 Department of Genetics, Physiology and Microbiology, Faculty of Biological Sciences, Universidad Complutense de Madrid, Av. Complutense, 28040 Madrid, Spain; 3 Department of Neurology, Hospital Universitario Ramón y Cajal, IRYCIS, Ctra. Colmenar km 9.1, 28034 Madrid, Spain; rocio.vera@salud.madrid.org (R.V.-L.); acruzc@salud.madrid.org (A.C.-C.); jaime.masjuan@salud.madrid.org (J.M.); 4 Department of Medicine, Facultad de Medicina, Universidad de Alcalá, Ctra. Madrid-Barcelona km 33.6, 28871 Alcalá de Henares, Spain

**Keywords:** cerebral ischemia, eIF4E, eIF4G1, protein synthesis regulation, protein phosphorylation, neuronal death, vulnerable regions, confocal microscopy

## Abstract

Ischemic strokes are caused by a reduction in cerebral blood flow and both the ischemic period and subsequent reperfusion induce brain injury, with different tissue damage depending on the severity of the ischemic insult, its duration, and the particular areas of the brain affected. In those areas vulnerable to cerebral ischemia, the inhibition of protein translation is an essential process of the cellular response leading to delayed neuronal death. In particular, translation initiation is rate-limiting for protein synthesis and the eukaryotic initiation factor (eIF) 4F complex is indispensable for cap-dependent protein translation. In the eIF4F complex, eIF4G is a scaffolding protein that provides docking sites for the assembly of eIF4A and eIF4E, binding to the cap structure of the mRNA and stabilizing all proteins of the complex. The eIF4F complex constituents, eIF4A, eIF4E, and eIF4G, participate in translation regulation by their phosphorylation at specific sites under cellular stress conditions, modulating the activity of the cap-binding complex and protein translation. This work investigates the phosphorylation of eIF4G1 involved in the eIF4E/eIF4G1 association complex, and their regulation in ischemia-reperfusion (IR) as a stress-inducing condition. IR was induced in an animal model of transient cerebral ischemia and the results were studied in the resistant cortical region and in the vulnerable hippocampal CA1 region. The presented data demonstrate the phosphorylation of eIF4G1 at Ser^1147^, Ser^1185^, and Ser^1231^ in both brain regions and in control and ischemic conditions, being the phosphorylation of eIF4G1 at Ser^1147^ the only one found in the eIF4E/eIF4G association complex from the cap-containing matrix (m^7^GTP-Sepharose). In addition, our work reveals the specific modulation of the phosphorylation of eIF4G1 at Ser^1147^ in the vulnerable region, with increased levels and colocalization with eIF4E in response to IR. These findings contribute to elucidate the molecular mechanism of protein translation regulation that underlies in the balance of cell survival/death during pathophysiological stress, such as cerebral ischemia.

## 1. Introduction

A temporary or permanent reduction in blood flow to the brain can limit the availability of oxygen and nutrients to a particular region or to the entire brain, causing a cerebrovascular disease or stroke. Worldwide, a stroke is the second most common cause of death and a major origin of long term disability in adults [1,2,3]. Briefly, during brain ischemia, the lack of oxygen induces ATP depletion and impaired mitochondrial function. As a result, several cellular mechanisms are disrupted, leading to a progressive neuronal death in a biochemical sequence of events called ischemic cascade [4]. When blood flow is restored, blood reperfusion renews the supply of oxygen and nutrients to the ischemic areas, leading to the recovery of cellular function, but also inducing oxidative stress and the activation of multiple adverse biochemical mechanisms, producing a secondary neuronal death [5,6]. During ischemia, protein translation is suppressed by ATP depletion, but on reperfusion, protein synthesis recovers almost completely in most regions of the brain, referred to as ischemia-resistant regions. However, translation remains repressed in some regions, named of ‘ischemic vulnerability’, such as the hippocampal *cornu ammonis* 1 (CA1) region, where the translation machinery is never restored [7,8,9,10]. Vulnerable brain regions would be deficient in proteins essential for cell survival, leading to an irreversible loss of cell viability. Therefore, inhibition of protein synthesis is a specific parameter that corresponds to cell death after ischemia-reperfusion (IR) stress [9,10,11,12].

Cellular protein synthesis is generally determined by a rate-limiting stage, the initiation of translation. The initiation step is a highly regulated mechanism with an important checkpoint in the 40S ribosomal subunit recruitment to the 5′ end of a specific mRNA [13,14]. In summary, all nuclear-encoded mRNAs in eukaryotes contain a modified 5′ end, the so-called ‘cap’ structure: m7GpppN (7-methylguanosine triphosphate, where N is any nucleotide); and a 3′ polyadenylate (poly(A)) tail [15]. During cap-dependent initiation, the eukaryotic initiation factor (eIF) 4F complex—including the ATP-dependent RNA helicase eIF4A; eIF4E, which binds to the mRNA 5′-cap structure; and the scaffold protein eIF4G, which also interacts with the multi-protein eIF3 and with the poly(A)-binding protein (PABP)—facilitates the recruitment of a specific mRNA to form the 43S pre-initiation complex and initiates protein translation [13,16]. For this typical 5′-cap dependent translation, responsible for 90% of cellular proteins [17], eIF4E and eIF4G play an important role as key initiators of the eIF4F complex assembly [18].

The scaffolding protein eIF4G consists of three protein family members, eIF4G1 (referred as eIF4GI)—the main form with the highest expression—eIF4G3 (also named as eIF4GII)—a minor form with the lowest expression—and eIF4G2 (also known as p97, DAP5 or NAT1), which share homology with eIF4G1 and eIF4G3 in the middle and C-terminal regions [19,20]. eIF4G1 and eIF4G3 have a N-terminal region for the binding of PABP and eIF4E, in contrast with eIF4G2, which cannot interact due to the lack of the N-terminal domain [21,22]. eIF4Gs are phosphoproteins and eIF4G1 is phosphorylated by several serine/threonine-protein kinases, including the mammalian target of rapamycin (mTOR), activated by the phosphoinositide 3-kinase (PI3K)–protein kinase B (PKB or Akt) signaling pathway [23], which as mTOR complex 1 (mTORC1) responds to growth factors, stress, and energy status of the cells [24]. This first study of eIF4G1 phosphorylation [23] identified three major phospho-sites as molecular targets of mTOR signaling pathway, corresponding to Ser^1108^, Ser^1148^, and Ser^1192^; equivalent positions to Ser^1147^, Ser^1187^, and Ser^1231^, respectively, in the human canonical sequence of eIF4G1 (Q04637-1, UniProt database, https://www.uniprot.org/, accessed on 16 December 2021), sequence referred from here on. In addition, some reports have confirmed that an intense inhibition of mTOR activity occurs as a consequence of ischemic brain damage [25,26,27,28]. Remarkably, the Ser^1231^ site is a multitarget residue that can be also phosphorylated by the extracellular signal-regulated kinase 1 and 2 (ERK1/2) [29]—whose expression and activation are enhanced upon transient cerebral ischemia [30]—and additionally, by the cyclin-dependent kinase 1 (Cdk1) [31,32]. Finally, another serine described is Ser^1185^, regulated by protein kinase C alpha (PKCα) [31], protein kinase related to damage in transient global ischemia [33]. Despite all the knowledge regarding eIF4G phosphorylation sites, the specific function or consequence of the phosphorylation of eIF4G1 in translation has not been described and is not well established.

With all these phosphorylation regulation sites reported, we decided to study Ser^1147^, Ser^1185^, and Ser^1231^ phosphorylation sites as three different targets of the mTOR, PKCa, and ERK1/2 signaling pathways, respectively, related with ischemic stress. The aim of this study was to investigate the changes in these phosphorylations in response to IR stress, by analyzing their consequences in the eIF4E/eIF4G1 association found in resistant and vulnerable brain regions following IR damage. The presented data demonstrate the phosphorylation of eIF4G1 at Ser^1147^, Ser^1185^, and Ser^1231^ in brain regions both in control and ischemic conditions, being the phosphorylation of eIF4G1 at Ser^1147^ the only one found in the eIF4E/eIF4G association complex from the cap-containing matrix (m^7^GTP-Sepharose). In addition, our work reveals the specific modulation of the phosphorylation of eIF4G1 at Ser^1147^ in the hippocampal CA1 region, with increased levels and colocalization with eIF4E in this vulnerable brain region in response to ischemia-reperfusion.

## 2. Results

### 2.1. Rat eIF4G1 Sequence Reports Phosphorylation Sites Homologous to Human eIF4G1 in the IDL Region

**Figure 1:**
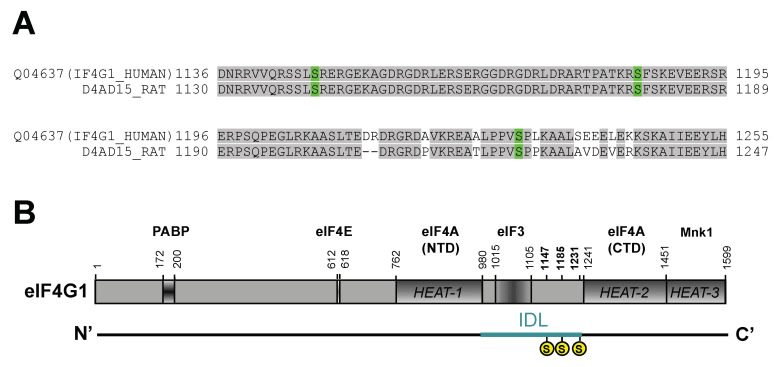
Phosphorylation sites in eIF4G1 sequence. (**A**) Alignment of human and rat eIF4G1 sequences in the region of the phosphorylation sites studied in this work. Amino acid positions with homology between human and rat are shaded in grey and serine phospho-sites studied are marked in green. (**B**) eIF4G1 protein domains. Serine phospho-sites studied are marked in yellow in the interdomain linker (IDL) region. Labels of initiation factors (eIFs) and other proteins indicate the binding region to eIF4G1.

To explore the post-translational modification of eIF4G1 by protein phosphorylation, we analyzed the main specific phosphorylation region of eIF4G1 described in the human amino acid sequence, and then we compared the sequence of eIF4G1 in humans and rats with bioinformatics tools. Our sequence-based alignment revealed that this region is highly conserved and that rat eIF4G1 is a counterpart to human eIF4G1 (Figure 1A). Thus, the specific phosphorylation sites of interest, Ser^1147^, Ser^1185^, and Ser^1231^ in the human eIF4G1 sequence [23,29,31], corresponded to Ser^1140^, Ser^1178^, and Ser^1222^ in the rat eIF4G1 sequence, respectively, and we could consider them as potential phospho-regulatory sites of interest to study under IR stress in our experimental model. Moreover, these eIF4G1 phospho-sites were localized in the interdomain linker (IDL), a dynamic region described in between HEAT (Huntingtin, elongation factor 3, a subunit of protein phosphatase 2A, and target of rapamycin) domains 1 and 2 of eIF4G1, enriched in phosphorylation sites that respond to cell stimulation signaling [29,31] (Figure 1B). Hereinafter, in this paper we will use the serine position of the human eIF4G1 sequence to designate the rat eIF4G1 phospho-serine sites studied.

### 2.2. eIF4G1 Shows a Similar Level of Expression in the Cerebral Cortex and Hippocampal CA1 Region under Ischemia-Reperfusion (IR) Stress

**Figure 2:**
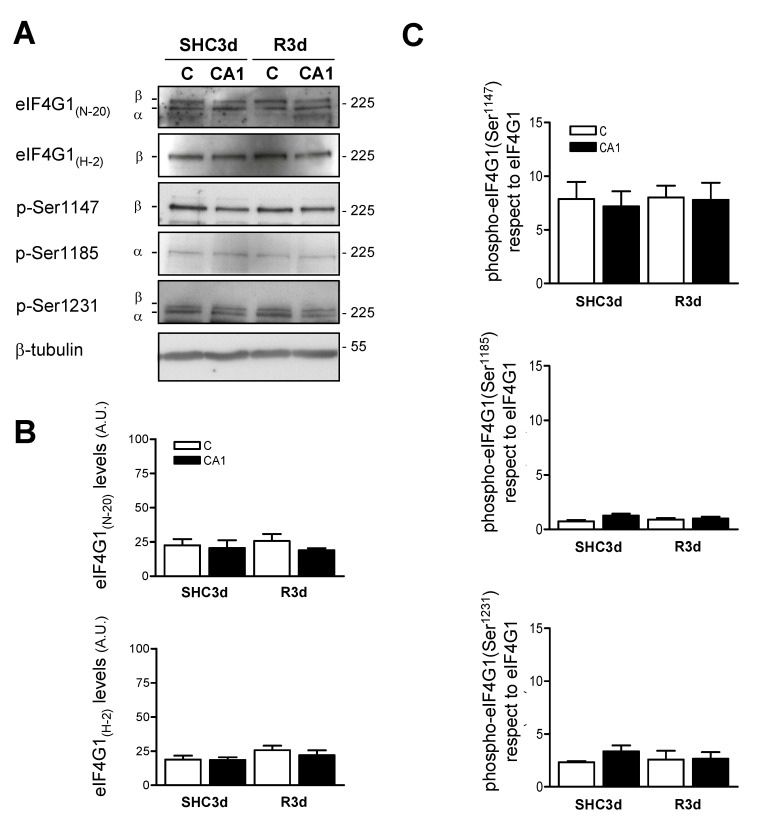
Identification of eIF4G1 phosphorylation sites induced by ischemia-reperfusion (IR) stress. (**A**) Samples of the cerebral cortex (C) or hippocampal CA1 region from control (SHC3d) and ischemic animals with reperfusion (R3d), were analyzed by Western blotting with anti-eIF4G1 N-20 (eIF4G1_(N-20)_), anti-eIF4G1 H-2 (eIF4G1_(H-2)_), anti-phospho-eIF4G1 Ser^1147^ (p-Ser1147), anti-phospho-eIF4G1 Ser^1185^ (p-Ser1185), anti-phospho-eIF4G1 Ser^1231^ (p-Ser1231), and anti-β-tubulin (β-tubulin) antibodies. The α and β forms of eIF4G1 were indicated; numbers on the right indicate the apparent molecular mass in kDa from protein markers. The figures are representative results of 4–6 independent experiments from 4–6 animals. Full original images of the Western blots are shown in the Supplementary Material (Figure S2) (**B**) Quantification of the eIF4G1 levels in Western blots using eIF4G1_N-20_ (upper) or eIF4G1_H-2_ (lower) antibodies. (**C**) Quantification of eIF4G1 phosphorylated (phospho-eIF4G1) at Ser^1147^, Ser^1185^ and Ser^1231^ residues with respect to total eIF4G1 levels (ratios) detected with anti–eIF4G1_(N-20)_ antibody. Bar graphs represent the mean of 4–6 independent experiments from 4–6 animals; error bars indicate SE. No statistical significance was found.

Prior to the study of the phosphorylation status of eIF4G1 under IR stress, we firstly analyzed the protein expression in brain samples from the ischemia model by Western blotting. We studied the cerebral cortex and hippocampal CA1 region as characteristic resistant and vulnerable regions to ischemia, respectively, in both control and ischemic animals. eIF4G1 was detected with anti-eIF4G1 N-20 antibody identifying two isoforms, α and β, according to their electrophoretic mobility (Figure 2A). Using the anti-eIF4G1 H-2 antibody, only the band corresponding to β isoform was detected. The eIF4G1 protein levels detected showed equal expression in control and ischemic condition, and they did not show significant changes between the brain regions studied, with no differences between the cortical and CA1 regions in both control (SHC3d) and ischemic (R3d) samples (Figure 2B). These results demonstrate that eIF4G1 levels were not affected by IR stress, including the translational inhibition state existent in the ischemia-vulnerable CA1 region [11].

### 2.3. Characterization of the Phosphorylation Status of eIF4G1 at Ser^1147^, Ser^1185^, and Ser^1231^ Phospho-Sites in the Cortical and CA1 Brain Regions

We analyzed these phosphorylation sites in SHC3d control and R3d ischemic samples, and detected the phosphorylation of eIF4G1 at Ser^1147^, Ser^1185^, and Ser^1231^, finding that all of them had the same levels in the cortical and CA1 regions, without significant changes between these regions (Figure 2A). Additionally, our data did not show significant changes in the R3d condition compared with its respective SHC3d control (Figure 2C). Despite not having detected changes, the presence of these phosphorylations in vivo can open a new path of knowledge in the eIF4E/eIF4G1 complex regulation, and, therefore, in the protein synthesis response under a physio-pathological condition, such as IR stress. 

### 2.4. eIF4G1 Bound to eIF4E is Phosphorylated at Ser^1147^

**Figure 3:**
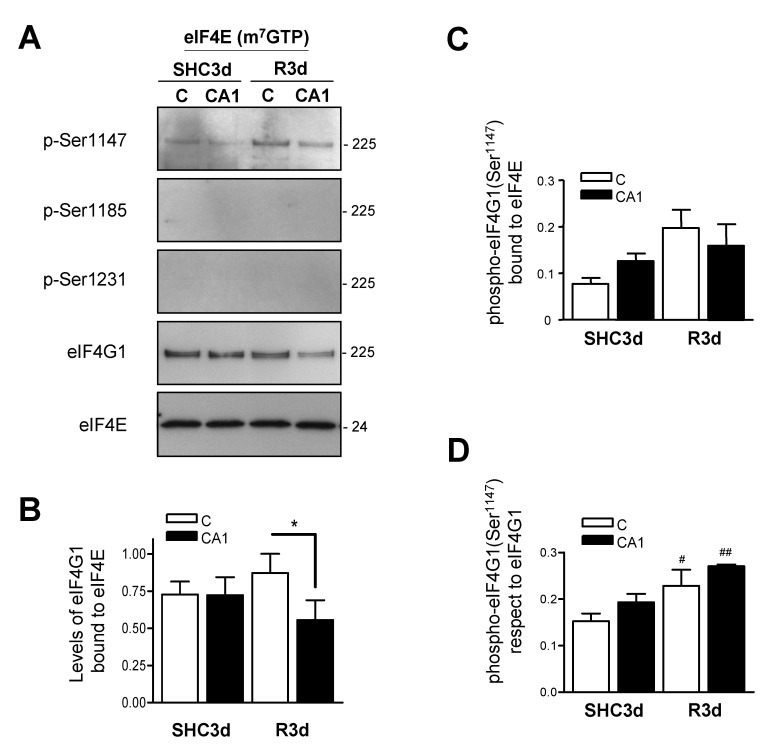
eIF4G1 phosphorylated at Ser^1147^ is bound to eIF4E. (**A**) Samples of cerebral cortex (C) or hippocampal CA1 region from control (SHC3d) and ischemic animals with reperfusion (R3d), were bound to a cap-containing matrix (m^7^GTP-Sepharose) and eIF4E and eIF4E-associated proteins were analyzed by SDS-PAGE followed by Western blotting for anti-phospho-eIF4G1 Ser^1147^ (p-Ser1147), anti-phospho-eIF4G1 Ser^1185^ (p-Ser1185), anti-phospho-eIF4G1 Ser^1231^ (p-Ser1231), anti-eIF4G1_(N-20)_ (eIF4G1), and anti-eIF4E (eIF4E) antibodies. Among phospho-eIF4G1 forms, only eIF4G1 phosphorylated at Ser^1147^ was detected. Molecular mass (kDa) of protein markers is stated in the right. The figures are representative results of 4–6 independent experiments from 4–6 animals. Full original images of the Western blots are shown in the Supplementary Material (Figure S3) (**B**) Quantification of eIF4G1 bound to eIF4E, and (**C**), quantification of eIF4G1 phosphorylated at Ser^1147^ bound to eIF4E with respect to eIF4E levels (ratios). (**D**) Relative levels of eIF4G1 phosphorylated at Ser^1147^ bound to eIF4E with respect to eIFG1 levels. In all experiments, eIF4E was detected with anti-eIF4E antibody, and no significant differences between eIF4E levels were found. Bar graphs represent the mean of 4–6 independent experiments from 4–6 animals; error bars indicate SE. Statistical significance were performed by Newman–Keuls post-test (# *p* < 0.05; ## *p* < 0.01) or by Student’s *t*-test (* *p* < 0.05), after significant ANOVA (*p* < 0.05), compared with their respective control, or between the cerebral cortex and CA1 samples (indicated by lines).

To determine whether eIF4G1 phosphorylated is bound to eIF4E, we analyzed the phosphorylation status of eIF4G1 in eIF4E/eIF4G1 complexes by a pull-down assay of eIF4E with the cap-containing matrix m^7^GTP-Sepharose (Figure 3A). The results showed higher levels of eIF4G1 bound to eIF4E in the ischemic-resistant cortical region and, conversely, decreased levels in the ischemic-vulnerable CA1 region after IR stress (Figure 3B), as previously described [28]. Interestingly, only eIF4G1 phosphorylated at Ser^1147^ was found in the m^7^GTP-bound eIF4E fraction (Figure 3A), and it was increased in the eIF4E/eIF4G1 complex in both cortical and CA1 regions in IR condition compared to the control (Figure 3C), an increase that was significant with respect to eIF4G1 bound to eIF4E (Figure 3D). Furthermore, we did not observe eIF4G1 phosphorylated at Ser^1185^ or Ser^1231^ (Figure 3A). These results demonstrated that only eIF4G1 phosphorylated at Ser^1147^ is associated with eIF4E, suggesting a relevant participation of this phospho-site in the brain, phospho-site that was sensitive to IR stress. 

### 2.5. Association of eIF4E to eIF4G1 Phosphorylated at Ser^1147^ in eIF4G1 Immunoprecipitates

**Figure 4:**
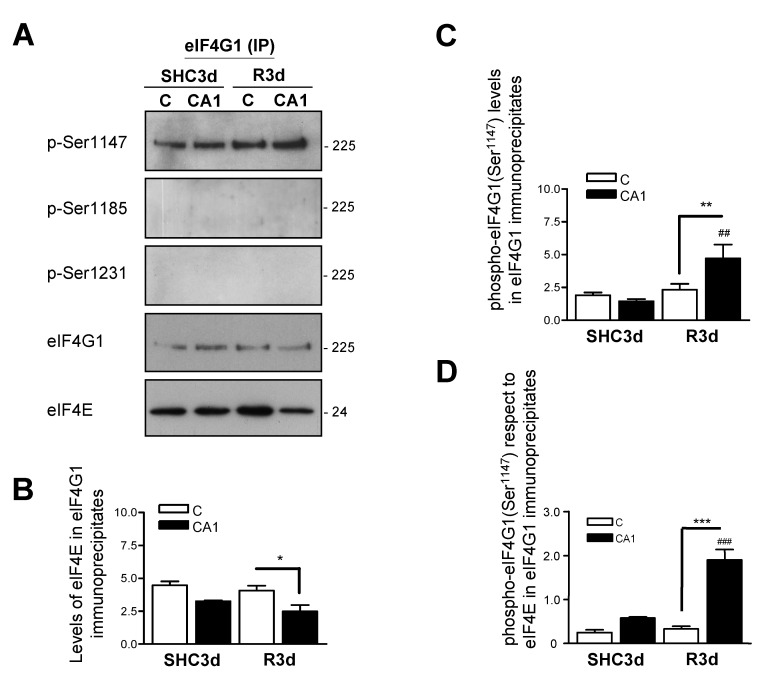
Association of eIF4E to eIF4G1 phosphorylated at Ser^1147^ in eIF4G1 immunoprecipitates. (**A**) Samples of cerebral cortex (C) or hippocampal CA1 region from control (SHC3d) and ischemic animals with reperfusion (R3d), were immunoprecipitated with anti–eIF4G1_(N-20)_ antibody and eIF4G1-associated proteins analyzed by SDS-PAGE followed by Western blotting for anti-phospho-eIF4G1 Ser^1147^ (p-Ser1147), anti-phospho-eIF4G1 Ser^1185^ (p-Ser1185), anti-phospho-eIF4G1 Ser^1231^ (p-Ser1231), anti-eIF4G1_(H-2)_ (eIF4G1) and anti-eIF4E (eIF4E) antibodies. Among eIF4G1 phospho-forms, only eIF4G1 phosphorylated at Ser^1147^ was detected. Numbers on the right indicate the apparent molecular mass in kDa, from protein markers. The figures are representative results of 4–6 independent experiments from 4–6 animals. Full original images of the Western blots are shown in the Supplementary Material (Figure S4). (**B**) Quantification of eIF4E levels, and (**C**) quantification of eIF4G1 phosphorylated at Ser^1147^, with respect to immunoprecipitated eIF4G1 levels (ratios). (**D**) Relative levels of eIF4G1 phosphorylated at Ser^1147^ with respect to eIF4E levels in eIF4G1 immunoprecipitates. Bar graphs represent the mean of 4–6 independent experiments from 4–6 animals; error bars indicate SE. Statistical significance were performed by Newman–Keuls post-test (## *p* < 0.01; ### *p* < 0.001) or by Student’s *t*-test (* *p* < 0.05; ** *p* < 0.01; *** *p* < 0.001), after significant ANOVA (*p* < 0.05), compared with their respective control, or between the cerebral cortex and CA1 samples (indicated by lines). IP, immunoprecipitated fraction.

To confirm the above result, we performed eIF4G1 immunoprecipitations in the cerebral cortex and CA1 samples from control and IR animals and detected the phosphorylation status of eIF4G1 and the associated eIF4E (Figure 4A). The results showed higher levels of eIF4E in eIF4G1 immunoprecipitates in the cerebral cortex, and a significant decrease in eIF4E in the CA1 region in ischemic R3d animals (Figure 4B). Moreover, the results showed phosphorylation of eIF4G1 only at Ser^1147^, while phosphorylation was not detected at Ser^1185^ or Ser^1231^ under any conditions (Figure 4A). In addition, the phosphorylation at Ser^1147^ was significantly increased in the CA1 region in R3d condition with respect to the cerebral cortex, an increase that was also significant compared with their control SHC3d (Figure 4C). Since eIF4E is present in the immunoprecipitated eIF4G1 (Figure 4B) it would be “active” eIF4E for protein translation—i.e., eIF4E associated with eIF4G1 when forming an active eIF4F complex—we calculated the levels of eIF4G1 phosphorylated at Ser^1147^ respect to eIF4E in eIF4G1 immunoprecipitates, as the “active” fraction of eIF4G1 in these immunoprecipitates (Figure 4D). This relative amount showed significantly higher levels of eIF4G1 phosphorylated at Ser^1147^ in the CA1 region compared with the cerebral cortex in ischemic R3d animals and compared with their control SHC3d (Figure 4D). Taking these results together, we found that, in brain tissue, a proportion of eIF4G1—i.e., phosphorylated at Ser^1185^ or Ser^1231^—was not bound to eIF4E, with unknown translation-related function or protein association, whereas eIF4G1 phosphorylated at Ser^1147^ was the phospho-form present in the eIF4E/eIF4G1 association complex. Interestingly, but contradictory, a reduction in the eIF4E/eIF4G1 complex in the CA1 region in R3d samples (Figure 3B and Figure 4B) was associated with a relative increase in the phosphorylation at Ser^1147^ in the eIF4G1 bound to eIF4E (Figure 3D and Figure 4D), the brain region that was vulnerable to ischemia and where translation inhibition was not recovered after IR [11].

### 2.6. Increased Colocalization of eIF4G1 Phosphorylated at Ser^1147^ with eIF4E in the CA1 Region under Ischemia-Reperfusion (IR) Stress

**Figure 5:**
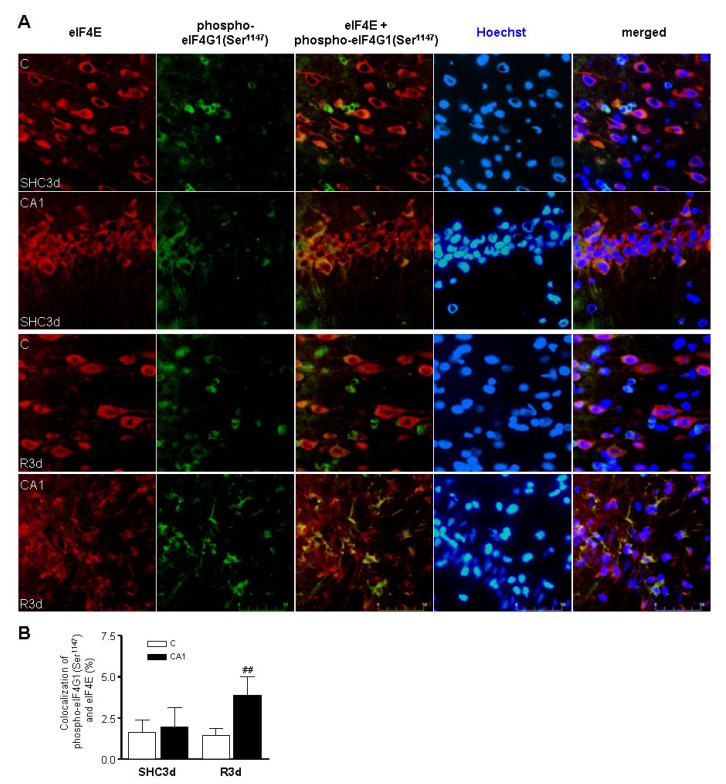
Colocalization of eIF4G1 phosphorylated at Ser^1147^ and eIF4E in the cerebral cortex and hippocampal CA1 regions induced by ischemia-reperfusion stress. (**A**) Brain sections of cerebral cortex (C) or hippocampal CA1 region from control (SHC3d) and ischemic animals with reperfusion (R3d), were used for eIF4E and eIF4G1 phosphorylated at Ser^1147^ colocalization study by confocal fluorescence microscopy. eIF4E was visualized using Alexa Fluor 568 secondary antibody (red) while phospho-eIF4G1 at Ser^1147^ was visualized with Alexa Fluor 488 secondary antibody (green). Cell nuclei were stained with Hoechst 33342 dye (blue). Green and red channels were merged and colocalized components are shown in yellow (eIF4E +phospho-eIF4G1, central images). Merged images display the eIF4E (red), phospho-eIF4G1 (green) and Hoechst (blue) signal. Images are representative results from four to six different animals and are full original scanned images. Scale bar, 50 μm. (**B**) Quantification of eIF4G1 phosphorylated at Ser^1147^ and eIF4E colocalization. The degree of colocalization is expressed by the percentage of green objects colocalizing with red objects in the scanned area. Data are from four to six different animals; error bars indicate SE. ## *p* < 0.01 by Newman–Keuls post-test after significant ANOVA (*p* < 0.05), compared with their control and with the cerebral cortex.

To assess the above results, we analyzed the colocalization of eIF4G1 phosphorylated at Ser^1147^ and eIF4E by confocal fluorescence microscopy in cortical and CA1 brain sections from SHC3d control and R3d ischemic animals (Figure 5A). The images revealed a cytoplasmic colocalization of eIF4E and eIF4G1 phosphorylated at Ser^1147^ in the cortical and CA1 regions from SHC3d control and R3d sections, although a loss of cellular integrity could be observed in the CA1 region in R3d (Figure 5A). The cortical and CA1 regions can be observed as a whole in the stained brain sections shown in Supplementary Figure S5. Interestingly, after quantification of the colocalization between eIF4E and eIF4G1 phosphorylated at Ser^1147^, we detected significantly higher levels of colocalization in CA1 neurons in R3d ischemic animals (Figure 5B), confirming the results observed in the cap-containing matrix (m^7^GTP-Sepharose) and eIF4G1 immunoprecipitation assays. 

## 3. Discussion

There is a strong correlation between translation inhibition in vulnerable regions to ischemia, and ischemic-induced neuronal death [34]. The translation shut-off occurs during cerebral ischemia, mainly by the energy failure [9,35], and its restoration takes place with the recovery of the energy status in the reperfusion after ischemia. However, in some vulnerable brain regions, including the hippocampal CA1 region [36], translation never restarts, and a neuronal death occurs several days after ischemia, referred to as delayed neuronal death [37]. A key regulatory step responsible for the efficiency of translation initiation is the recruitment of a specific mRNA by the cap-binding eIF4F complex to form the 48S initiation complex [38]. In the eIF4F complex, the scaffold protein eIF4G, interacts directly with eIF4E and enables the assembly of the following initiation factors. It is established that the availability of eIF4E depending on the eIF4E/eIF4G association contributes to the progress of translation during cellular stress response. Thus, while the specific inhibition of eIF4E by eIF4E-binding proteins (4E-BPs) is a well-known control mechanism of translation inhibition, including cerebral ischemia [11,26,28,39,40,41], the knowledge of eIF4G regulation is incomplete.

Eukaryotic cells have two homolog isoforms of eIF4G, eIF4GI (eIF4G1), and eIF4GII (eIF4G3) that contain a similar C-terminal domain, eIF4G1 being the main form in mammalian cells [42]. The C-terminus comprises three HEAT domains of about 500 amino acids residues each [43], and an interdomain linker (IDL) divides HEAT domains 1 and 2. The IDL, analyzed by SMART domain software, has approximately 240 amino acids residues, ranging from amino acid positions 989 to 1242 in human eIF4G1, and from 983 to 1234 in rat eIF4G1. IDL provides numerous phosphorylation sites that can modulate dynamic protein–protein interactions in the central portion of eIF4G1 [29]. Using bioinformatics tools to compare human and rat eIF4G1 sequences, as well as published reports describing human phosphorylation of eIF4G1 sites [23,29,31,32], we selected three serine residues: Ser^1147^, Ser^1185^, and Ser^1231^, belonging to the IDL domain, that could be phosphorylated by three different signaling pathways, mTOR, PKCα, and ERK1/2, respectively, that are related to ischemic stress. These phospho-sites have a precise sequence alignment when comparing human and rat eIF4G1, and are specifically recognized by the same anti-phospho antibodies. It is worthwhile to note that the selected phospho-sites can differ by one amino acid in the sequence of published reports [23,29,31,32], this mismatch is due to a human eIF4G1 sequence with 1600 amino acids, instead of the human eIF4G1 canonical sequence (Q04637-1) with 1599 amino acids used here.

In this report, we explore the hypothesis that phosphorylation of eIF4G1 can be a differential control mechanism between brain regions that are resistant and vulnerable to ischemia. For this purpose, we studied whether the phosphorylation of eIF4G1 could operate in the eIF4E/eIF4G association complex as a regulatory mechanism in the translation inhibition induced by ischemia-reperfusion, analyzing their phosphorylation status in SHC3d control and R3d ischemic samples of the cortical resistant and CA1 vulnerable regions. This study has been conducted in ischemic young adult animals, although aging and co-morbidities are important determinants of outcome after an ischemic stroke. Since this study investigates a molecular mechanism in brain regions vulnerable to ischemia, the inclusion of a co-morbidity in the study would mean that the molecular mechanism could be associated with processes related to co-morbidity, rather than with a precise knowledge of the ischemic pathophysiology. In addition, the results obtained may be specific to the co-morbidity studied and exhibit new effects in a different co-morbidity. Therefore, co-morbidity has been avoided in this study. From the results achieved in this report, several novel findings can be concluded: (i) phosphorylation of eIF4G1 at Ser^1147^, Ser^1185^, and Ser^1231^ is present in brain tissue, without changes between the different regions studied nor between the control or the ischemic condition; (ii) among the three phospho-sites studied, the phosphorylation of eIF4G1 at Ser^1147^ was the only one found in the eIF4E/eIF4G association complex from the cap-containing matrix (m^7^GTP-Sepharose); (iii) phosphorylation of eIF4G1 at Ser^1147^ is increased in the vulnerable CA1 region in response to IR stress, as demonstrated eIF4G1 immunoprecipitation assays; and (iv) phosphorylation of eIF4G1 at Ser^1147^ is colocalized with eIF4E in brain sections, with higher levels in the CA1 region after IR.

Several studies have shown different signaling pathways involved in phosphorylation of eIF4G1 [23,29,31,32], but there is a lack of knowledge about the function or consequences of these phosphorylations. The PI3K–mTORC1 signaling pathway is a crucial regulatory signaling for translation activation in response to growth factors, an adequate nutritional cellular status or the energy status of the cells [24,27,44,45]. The mTOR protein kinase phosphorylates and inactivates 4E-BPs [27,46], releasing active eIF4E for protein translation, and, either by direct or indirect phosphorylation, renders eIF4G1 phosphorylated at Ser^1147^, Ser^1187^, and Ser^1231^ in response to serum-stimulation, resulting in a fully active eIF4G1 [23]. In addition, PKCα and ERK1/2 phosphorylate eIF4G1 at Ser^1185^ and Ser^1231^, respectively, in a mitogenic signal transduction, inducing a rearrangement of the complex with eIF4A/eIF4B/eIF3 and increasing the mitogen-activated protein kinase-interacting kinase Mnk1 association [29,31,47]. In our work, phosphorylation of eIF4G1 at Ser^1147^, Ser^1185^, and Ser^1231^ was detected in both hippocampal and cortical brain regions, in control and ischemic conditions, although changes in these phospho-sites were not found under IR stress. Interestingly, while the role of phosphorylation of eIF4G1 is not well established [23,29,31,32], when we studied the eIF4E/eIF4G1 association by cap-containing matrix (m^7^GTP-Sepharose), eIF4G1 immunoprecipitation and eIF4E/phospho-eIF4G1 colocalization, only phosphorylation of eIF4G1 at Ser^1147^ was detected. Since the eIF4G1 present in the cap-containing matrix is bound to eIF4E, it means that it is “active” eIF4G1 for protein translation—i.e., eIF4G1 associated with eIF4E forming active eIF4F complex—and because it is only phosphorylated at Ser^1147^ (not on Ser^1185^ nor Ser^1231^), it can be concluded that this phosphorylation characterizes to “active” eIF4G1, even though the precise mechanism by which this phosphorylation may affect the activity of eIF4G1 is unknown. Consistent with different intracellular pools of eIF4G1, changes in intracellular compartmentalization of eIF4G and eIF4G1 phosphorylated at Ser^1147^ have been reported in different brain regions [48]. However, while this report links phosphorylation of eIF4G1 at Ser^1147^ with protein aggregates associated with translation inhibition, our results identify this phosphorylation in the eIF4E/eIF4G1 complex, active for protein translation, in agreement with other reports that associate this phosphorylation with increased translation [23,49]. 

A crucial mechanism for increasing the assembly of the eIF4E/eIF4G1 complex is to enhance the cellular availability of eIF4E to bind with eIF4G, a state controlled by 4E-BPs translation repressors [40]. The 4E-BPs (mainly 4E-BP2 in brain tissue) compete with eIF4G to bind eIF4E and alter the cellular distribution of eIF4E from a protein-translating eIF4E/eIF4G1 complex to a translation-inhibiting eIF4E/4E-BP2 complex. Herein we describe an increase in eIF4G1 phosphorylated at Ser^1147^ in the CA1 region under IR stress, phosphorylation that has been related to an active eIF4E/eIF4G1 complex assembly and with increased protein translation rates [23,49]. However, previous results showed that this region had significantly increased association of eIF4E to 4E-BP2 [11,28] and inhibition of protein synthesis with apoptosis triggering in the vulnerable CA1 region in response to ischemia-reperfusion [11]. The fact that eIF4E is more associated with 4E-BP2 in the CA1 region under IR stress, could result in that only the eIF4G1 with the highest affinity for eIF4E may be able to compete with 4E-BP2 and associate with eIF4E. Thus, the phosphorylation of eIF4G1 at Ser^1147^ may serve to enhance the recruitment of eIF4E in response to a stress situation—IR stress—and make feasible a minimal protein synthesis required, e.g., for programmed cell death mechanisms. In this sense, the results that show higher levels of eIF4G1 phosphorylated at Ser^1147^ after IR stress (Figure 3A,C), higher proportion of this phosphorylation respect to the levels of eIF4E associated with eIF4G1 (Figure 4D) and the colocalization between eIF4G1 phosphorylated at Ser^1147^ and eIF4E (Figure 5), support the proposed function for the phosphorylation of eIF4G1 at Ser^1147^. 

Overall, our results study three phosphorylation sites, Ser^1147^, Ser^1185^, and Ser^1231^, in the translation initiation factor eIF4G1 and their occurrence in the eIF4E/eIF4G1 complex during post-ischemic reperfusion in brain tissue. Current work demonstrates that only eIF4G phosphorylated at Ser^1147^ is found when it is associated with eIF4E, suggesting a more relevant role of this phospho-site in translation initiation. In addition, the vulnerable CA1 region to ischemia exhibits an increase in the levels of phosphorylation of eIF4G1 at Ser^1147^, with higher proportion of this phosphorylation respect to the levels of eIF4E associated with eIF4G1 and colocalized with eIF4E, in an environment of decreased eIF4E/eIF4G1 complex (this report) and translation inhibition where neuronal death occurs several days after ischemia [11,12]. This delayed neuronal death occurs in vulnerable regions to ischemia as a result of apoptosis progression, characterized by a general, but incomplete, inhibition of cap-dependent protein synthesis and some cap-independent translation [50,51,52]. Our results would be in agreement with this phenomenon: upon reperfusion after ischemic stress, selected neurons, e.g., those of the CA1 region, would have a resilient eIF4G1 (phosphorylated at Ser^1147^ to compete with 4E-BPs) bound to eIF4E and maintain a limited cap-dependent translation to progress in the apoptotic mechanism. To confirm the physiological role of eIF4G1 phosphorylation at Ser^1147^ in the initiation step of protein translation and elucidate whether this phosphorylation could be a target to recover brain regions vulnerable to ischemia, further studies would be needed to assess the implications of this phosphorylation in the survival/death balance in brain tissue under IR stress. 

## 4. Materials and Methods

### 4.1. Materials

All general products were purchased from Sigma-Aldrich (Merck KGaA, Darmstadt, Germany) except those indicated in the text. Chemicals used in the gel electrophoresis were obtained from Bio-Rad (Madrid, Spain) and Cytiva (formerly GE Healthcare, Barcelona, Spain). Rabbit polyclonal anti-phospho-eIF4G1 (Ser1108)—Ser1147 in the human canonical sequence Q04637-1 from UniProt, see above—antibody (#2441) was from Cell Signalling Technology (Beverly, MA, USA). Rabbit polyclonal anti-phospho-eIF4G1 (Ser1185) antibody (bs-4003R) was from Bioss (Woburn, MA, USA). Rabbit polyclonal anti-phospho-eIF4G1 (Ser1231) antibody (GTX79087) was from GeneTex (Irvine, CA, USA). Mouse monoclonal anti-eIF4E antibody (610269) was from BD Transduction Laboratories (BD Biosciences, Erembodegen, Belgium). Mouse monoclonal anti-eIF4G1 (H-2, sc-373892) and goat polyclonal anti-eIF4G1 (N-20, sc-9601) antibodies were from Santa Cruz Biotechnology (Santa Cruz, CA, USA). Mouse monoclonal anti-β-tubulin antibody (T5201) was from Sigma-Aldrich (Merck KGaA, Darmstadt, Germany). Secondary antibodies used for immunodetection were anti-mouse (NA931), -rabbit (NA934) (Cytiva, Barcelona, Spain), or -goat (sc-2020) IgG peroxidase-conjugated antibodies (Santa Cruz Biotechnology, Santa Cruz, CA, USA). Secondary antibodies used for immunohistochemistry were donkey anti-rabbit IgG (H+L) Alexa Fluor 488-conjugated antibody (A-21441) from Thermo Fisher Scientific (formerly Invitrogen, Waltham, MA, USA) and goat anti-mouse IgG (H+L) Alexa Fluor 568-conjugated antibody (A-11031) from Thermo Fisher Scientific (formerly Life Technologies, Waltham, MA, USA).

### 4.2. Sequence Analysis

The sequences of human and rat eIF4G1, Q04637 (IF4G1_HUMAN) and D4AD15 (D4AD15_RAT), respectively, were obtained from the UniProt database (https://www.uniprot.org/, accessed on 16 December 2021). Sequence alignment analysis of eIF4Gs was performed with pairwise bioinformatics tools (EMBOSS Needle) of EMBL-EBI (https://www.ebi.ac.uk/Tools/psa/emboss_needle/, accessed on 30 November 2021), prior to sequence similarities analysis performed by ESPript3.0 program (http://espript.ibcp.fr/ESPript/ESPript/, accessed on 30 November 2021) [53]. The analysis of conserved domains and regions of eIF4G was carried out with the SMART program (http://smart.embl-heidelberg.de/, accessed on 10 August 2021). 

### 4.3. Animal Model of Cerebral Ischemia and Reperfusion

Transient global forebrain ischemia was induced in adult male Wistar rats of 10–12 weeks of age (body weight, ~300 g) (Charles River, L’Arbresle, France) by the standard four-vessel occlusion (4VO) model, as described previously [28,54]. In brief, animals were anesthetized by intraperitoneal injection with 0.25 mg/kg atropine, 62.5 mg/kg ketamine, and 5 mg/kg diazepam, placed in a stereotaxic frame, and both vertebral arteries were permanently occluded by electrocoagulation. After 24 h, the animals were anesthetized with 4% isoflurane for induction and 2–2.5% isoflurane for maintenance (in 80% N_2_/20% O_2_) during the dissection of common carotid arteries, then ischemia was induced by carotid occlusion with atraumatic clips for 15 min. Next, the clips were removed to allow reperfusion (Supplementary Figure S1). The body temperature was maintained at 37 °C throughout the surgical procedure. After 3 days of reperfusion (R3d), animals were euthanized under deep anesthesia. Sham control animals (SHC3d) were performed as R3d animals without carotid occlusion. A total of 20 animals were used in this study, 10 animals for each SHC3d control and R3d group, which included the following experimental groups: cerebral cortex of SHC3d control animals; hippocampal CA1 region of SHC3d control animals; cerebral cortex of R3d ischemic animals; and hippocampal CA1 region of R3d ischemic animals (Figure S1). 

### 4.4. Sample Preparation

After euthanasia, the cerebral cortex and hippocampal CA1 region from both control and R3d ischemic animals were rapidly dissected and instantly frozen in dry ice for future procedures. The samples of brain regions were homogenized 1:5 (w/v) in buffer A (20 mM Tris-HCl, pH 7.5; 140 mM potassium chloride; 5 mM magnesium acetate; 1 mM dithiothreitol; 2 mM benzamidine; 1 mM EDTA; 2 mM EGTA; 10 µg/mL pepstatin A, leupeptin, and antipain; 20 mM sodium β-glycerophosphate; 20 mM sodium molybdate; 0.2 mM sodium orthovanadate), as previously described [25,55]. The tissue homogenates were then centrifuged at 12,000× *g* for 15 min to collect post-mitochondrial supernatant (PMS). All procedures were performed at 4 °C. The PMS fraction was kept at −80 °C until use, and protein concentrations in each sample were determined by Bradford assay (Bio-Rad, Madrid, Spain). For brain section preparations, SHC3d and R3d animals were sacrificed by intracardiac perfusion under deep anesthesia with 200 mL of saline with heparin through the left ventricle, followed by 4% paraformaldehyde (PFA) in phosphate-buffered saline (PBS) for brain fixation. After that, brains were removed and subsequently post-fixed by immersion in the same fixative solution overnight at 4 °C. The brains were then washed sequentially with 10%, 20%, and 30% sucrose/PBS (w/v), and included in Tissue-Tek O.C.T. (Sakura Finetek, Barcelona, Spain), before freezing at −80 °C.

### 4.5. eIF4G and eIF4E Binding Assay

To study eIF4G binding to eIF4E, a cap-containing matrix-7-methyl-GTP (m^7^GTP)-Sepharose (Cytiva, formerly GE Healthcare, Barcelona, Spain) was used, as described previously [26,28]. PMS samples (300 μg) for each experimental condition were added to 30 μL of 50/50 (w/v) suspension of m^7^GTP-Sepharose and incubated for 30 min at 4 °C in modified buffer A containing 100 mM potassium chloride and 100 μM GTP. The beads were centrifuged at 2500× *g* for 5 min and washed three times (5 min each), with the same buffer. Bound proteins were eluted from m^7^GTP-Sepharose with loading buffer (60 mM Tris-HCl, pH 6.8; 3% SDS; 2% β-mercaptoethanol; 5% glycerol; 0.0083% bromophenol blue) and analyzed by Western blotting after sodium dodecyl sulphate (SDS)-polyacrylamide gel electrophoresis (PAGE) (see below). 

### 4.6. Immunoprecipitation 

PMS samples (100 μg) were incubated with goat polyclonal anti-eIF4G antibody (1 μg) overnight, and then further incubated with Protein G-Agarose 4 (25 µL; 50% slurry, v/v in buffer A; ABT, Madrid, Spain) for 1 h at 4 °C on a rotary shaker [28]. The immunoprecipitates were recovered by centrifugation at 2500× *g* for 5 min, and were successively washed and centrifuged three times in buffer A. Finally, immunoprecipitated proteins were eluted from Protein G-Agarose with loading buffer (60 mM Tris-HCl, pH 6.8, 3% SDS, 2% β-mercaptoethanol, 5% glycerol, 0.0083% bromophenol blue) for SDS-PAGE and Western blotting (see below). Control experiments were performed in parallel using samples without anti-eIF4G antibody incubation.

### 4.7. Western Blot Analysis

PMS samples (35 μg), m^7^GTP-Sepharose-bound proteins, or eIF4G immunoprecipitates of each experimental condition were resolved by SDS-PAGE (10% acrylamide for PMS samples, or 7.5–15% acrylamide for m^7^GTP-Sepharose-bound and immunoprecipitated proteins; 3% cross-linking) (Cytiva, formerly GE Healthcare, Barcelona, Spain). Proteins were transferred onto PVDF membranes (Cytiva, Barcelona, Spain) and blocked (5% blocking agent, Cytiva, Barcelona, Spain) in 0.1 M PBS pH 7.4 for 1.5 h at room temperature. The membranes were then incubated for 1 h at room temperature or overnight at 4 °C with a primary antibody against the specific protein to be detected, washed three times (10 min each), and incubated for 1 h with peroxidase-conjugated anti-mouse, -rabbit or -goat IgG secondary antibody. Finally, blots were developed with the Clarity Western ECL Substrate reagent (Bio-Rad, Madrid, Spain) and ECL-Prime, or ECL-Select reagent (Cytiva, Barcelona, Spain). Detection of phosphoproteins was performed sequentially with phospho-specific primary antibodies and re-probed after stripping with a total-protein-specific primary antibody. The blots were quantified using Quantity One software (Bio-Rad, Madrid, Spain) with β-tubulin as internal standard. Protein levels were calculated in arbitrary units (A.U.) or expressed relative to total reference protein levels (ratios). Protein markers (range: 12–225 kDa) (Cytiva, Barcelona, Spain) were used to calculate the apparent molecular mass.

### 4.8. Immunohistochemistry 

Coronal brain sections, with the hippocampus region included, were prepared by cryostat sections at the interaural level +5.7 ± 0.2 mm on Real Capillary Gap microscope slides (Dako, Santa Clara, CA, USA), and were subsequently post-fixed with 4% PFA/PBS for 5 min at room temperature. After washing three times in PBS (5 min each), the sections were permeabilized with 10 mM sodium citrate pH 6.0 for 3 min at 95 °C, followed by cooling for 20 min at room temperature and washed in PBS for three times. Brain sections were then incubated with blocking solution (5% heat-inactivated donkey serum, 0.1% Triton X-100 in PBS) for 1 h at room temperature and incubated with primary antibodies overnight at 4 °C. After PBS washing three times, the sections were incubated with fluorochrome-conjugated secondary antibodies for 1 h at room temperature and washed three times again in PBS. Sections were mounted with coverslips in anti-fade solution containing p-phenylenediamine in glycerol-buffer and 30 μM bisbenzimide (Hoechst 33342) for nuclear staining. The fields of the cerebral cortex and hippocampal CA1 region of a given section were examined using a confocal laser-scanning microscope MRC-1024 (Bio-Rad, Madrid, Spain) at a wavelength excitation of 488 and 568 nm controlled by LaserSharp software (Bio-Rad, Madrid, Spain). The acquisition settings were kept constant for each image. Data acquisition was carried out sequentially to avoid interference with emission spectra between fluorochromes. Experiments with fluorochrome-conjugated secondary antibodies that omitted the primary antibody were used to check the background staining. Image acquisitions of more than 6 different fields and sections per sample were made with a 40x objective (1.8 zoom). LaserSharp software provides quantitative analysis of the degree of colocalization by evaluating the percentage of green objects that colocalize with red objects in the area of interest.

### 4.9. Statistical Analysis

Data are expressed as mean ± SE for the indicated number of experiments. Statistical significance between experimental groups was determined using the one-way ANOVA test and, when significant, it was followed by the Newman–Keuls post-test for multiple group comparisons, or the Student’s *t*-test for comparisons between the cerebral cortex and hippocampal CA1 region. All statistical analyses were performed with Prism software (GraphPad Software, San Diego, CA, USA) and the level of significance was established at α = 0.05.

## Supplementary Materials

The following are available online at https://www.mdpi.com/article/10.3390/ijms23031830/s1.
