# Supplementary material for: Phosphorylation of Eukaryotic Initiation Factor 4G1 (eIF4G1) at Ser1147 Is Specific for eIF4G1 Bound to eIF4E in Delayed Neuronal Death after Ischemia
Source: Int J Mol Sci. 2022 Feb 6;23(3):1830. doi: 10.3390/ijms23031830 (PMC8836865; doi:10.3390/ijms23031830)
Supplement: Supplementary file 1 [file ijms-23-01830-s001.zip › ijms-1558003-supplementary.pdf]

Supplementary Material

Phosphorylation of eukaryotic initiation factor 4G1 (eIF4G1) at Ser1147 is specific for eIF4G1 bound to eIF4E in delayed neuronal death after ischemia

Emma Martínez-Alonso [1,*], Natalia Guerra-Pérez [1,2,*], Alejandro Escobar-Peso [1], Lorena Peracho[1], Rocío Vera-Lechuga [3], Antonio Cruz-Culebras [3], Jaime Masjuan [3,4] and Alberto Alcázar [1,*]

[1] Department of Research, Hospital Universitario Ramón y Cajal, IRYCIS, Ctra. Colmenar km 9.1, 28034 Madrid, Spain; emma.martinez@hrc.es (E.M.-A.), alejandro.escobar@hrc.es (A.E.-P), lorena.peracho@salud.madrid.org (L.P.), alberto.alcazar@hrc.es (A.A.)

[2] Department of Genetics, Physiology and Microbiology, Faculty of Biological Sciences, Universidad Complutense de Madrid, Av. Complutense, 28040 Madrid, Spain; natalgue@ucm.es (N.G.-P)

[3] Department of Neurology, Hospital Universitario Ramón y Cajal, IRYCIS, Ctra. Colmenar km 9.1, 28034 Madrid, Spain; rocio.vera@salud.madrid.org (R.V.-L.), acruzc@salud.madrid.org (A.C.-C.), jaime.masjuan@salud.madrid.org (J.M.)

[4] Department of Medicine, Facultad de Medicina, Universidad de Alcalá, Ctra. Madrid-Barcelona km 33.6, 28871 Alcalá de Henares, Spain

* Correspondence: emma.martinez@hrc.es (E.M.-A.), natalgue@ucm.es (N.G.-P), alberto.alcazar@hrc.es (A.A.)

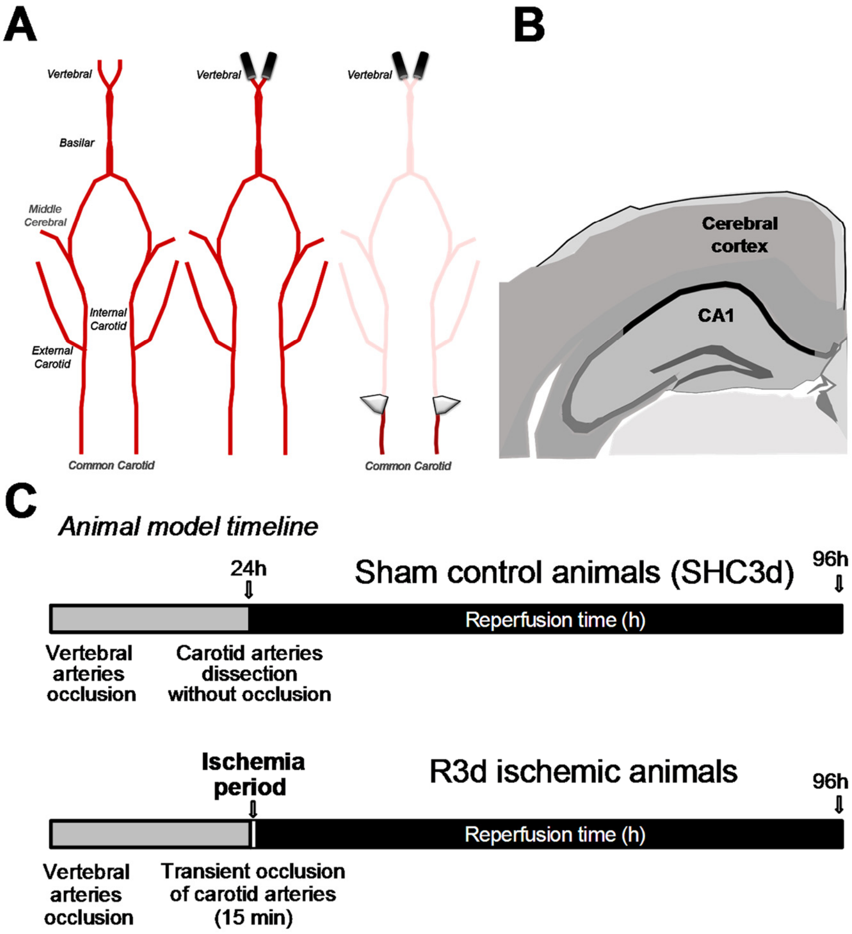

Figure S1. Animal model of transient cerebral ischemia. (**A**) Schematic representation of the cerebral arteries occluded in the ischemia model. Transient global cerebral ischemia was induced in adult rats by permanent occlusion of the vertebral arteries and, after 24h, transient occlusion of the common carotid arteries. (**B**) Representation of the brain regions used: the cerebral cortex and hippocampal *cornu ammonis* 1 (CA1) region. (**C**) Experimental model timeline in 3-day sham control animals (SHC3d) and in ischemic animals with 3 days of reperfusion (R3d).

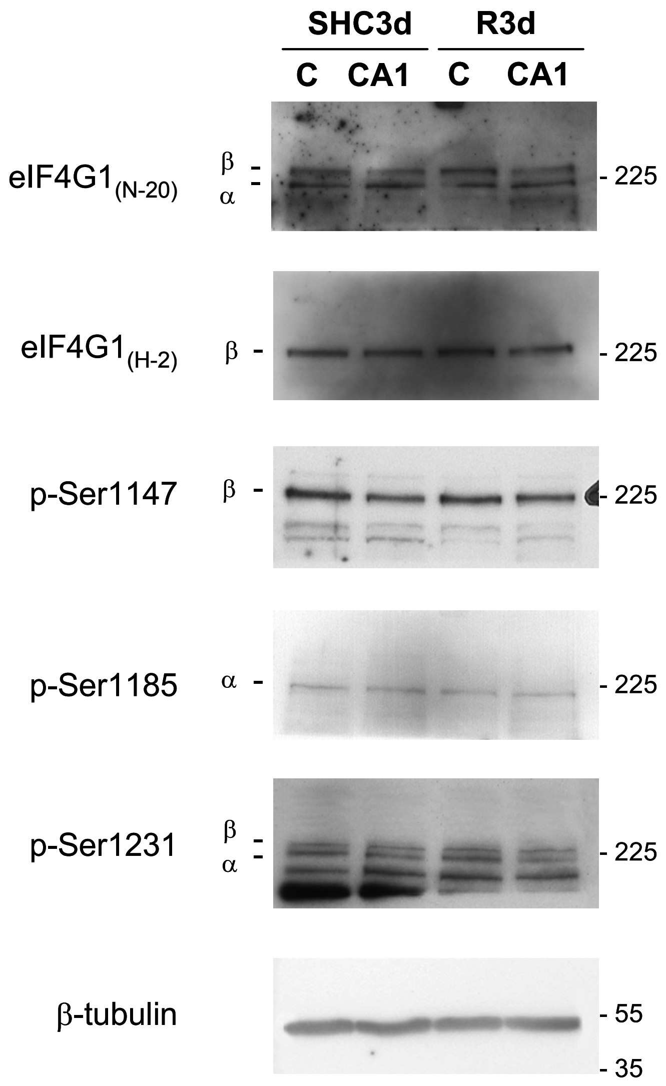

Figure S2. Full original images (uncropped) of Western blots of the Figure 2, incubated with anti–eIF4G1 N-20 (eIF4G1$_{(N-20)}$), anti–eIF4G1 H-2 (eIF4G1$_{(H-2)}$), anti–phospho-eIF4G1 Ser^{1147} (p-Ser1147), anti–phospho-eIF4G1 Ser^{1185} (p-Ser1185), anti–phospho-eIF4G1 Ser^{1231} (p-Ser1231) and anti–β-tubulin (β-tubulin) antibodies. Numbers on the right indicate the apparent molecular mass in kDa from protein markers.

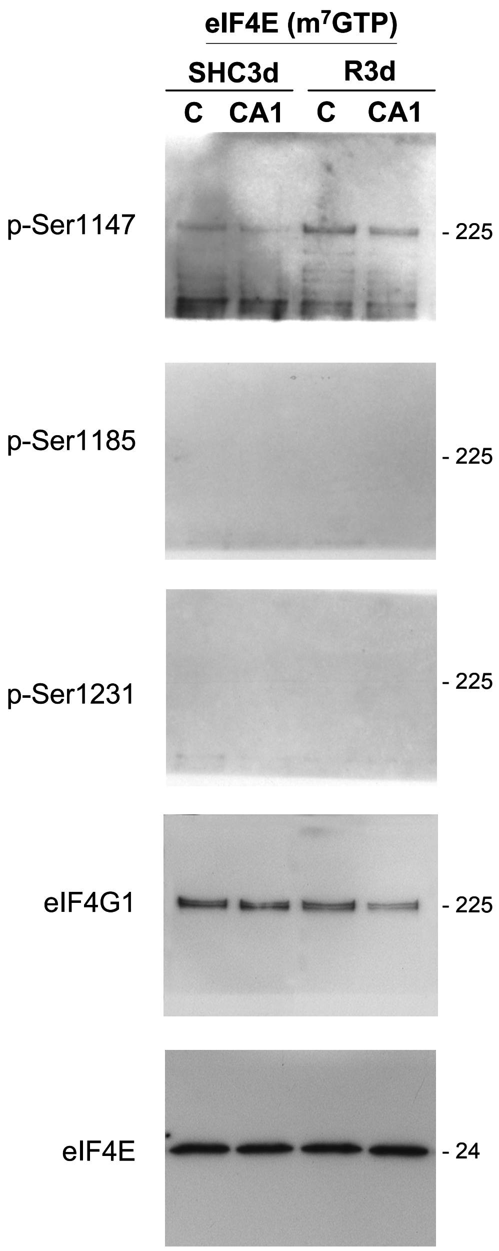

Figure S3. Full original images (uncropped) of Western blots of the Figure 3, incubated with anti–phospho-eIF4G1 Ser^{1147} (p-Ser1147), anti–phospho-eIF4G1 Ser^{1185} (p-Ser1185), anti–phospho-eIF4G1 Ser^{1231} (p-Ser1231), anti–$eIF4G1_{(N-20)}$ (eIF4G1) and anti–eIF4E (eIF4E) antibodies. Numbers on the right indicate the apparent molecular mass in kDa from protein markers.

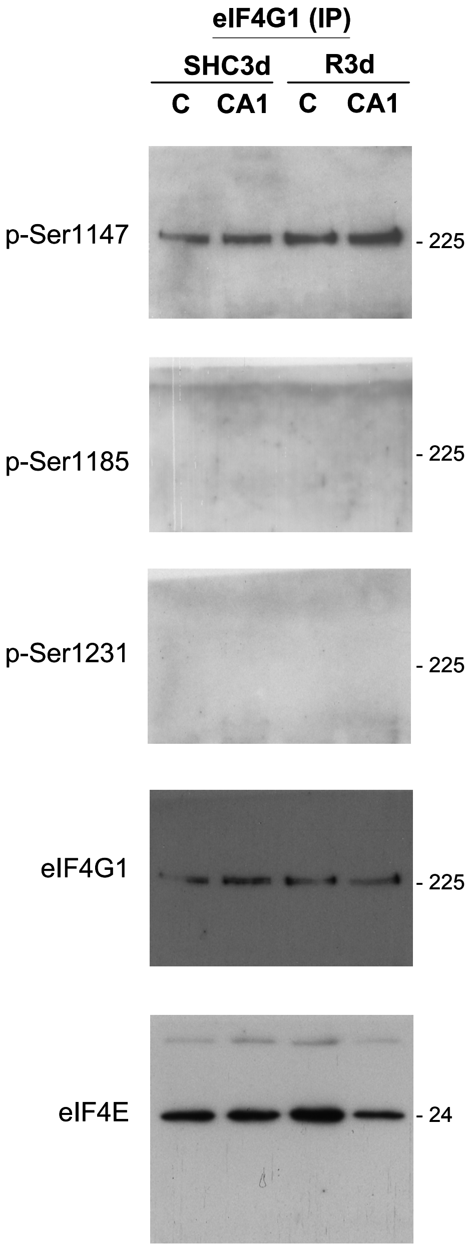

Figure S4. Full original images (uncropped) of Western blots of the Figure 4, incubated with anti–phospho-eIF4G1 Ser^{1147} (p-Ser1147), anti–phospho-eIF4G1 Ser^{1185} (p-Ser1185), anti–phospho-eIF4G1 Ser^{1231} (p-Ser1231), anti–$eIF4G1_{(H\text{-}2)}$ (eIF4G1) and anti–eIF4E (eIF4E) antibodies. Numbers on the right indicate the apparent molecular mass in kDa from protein markers.

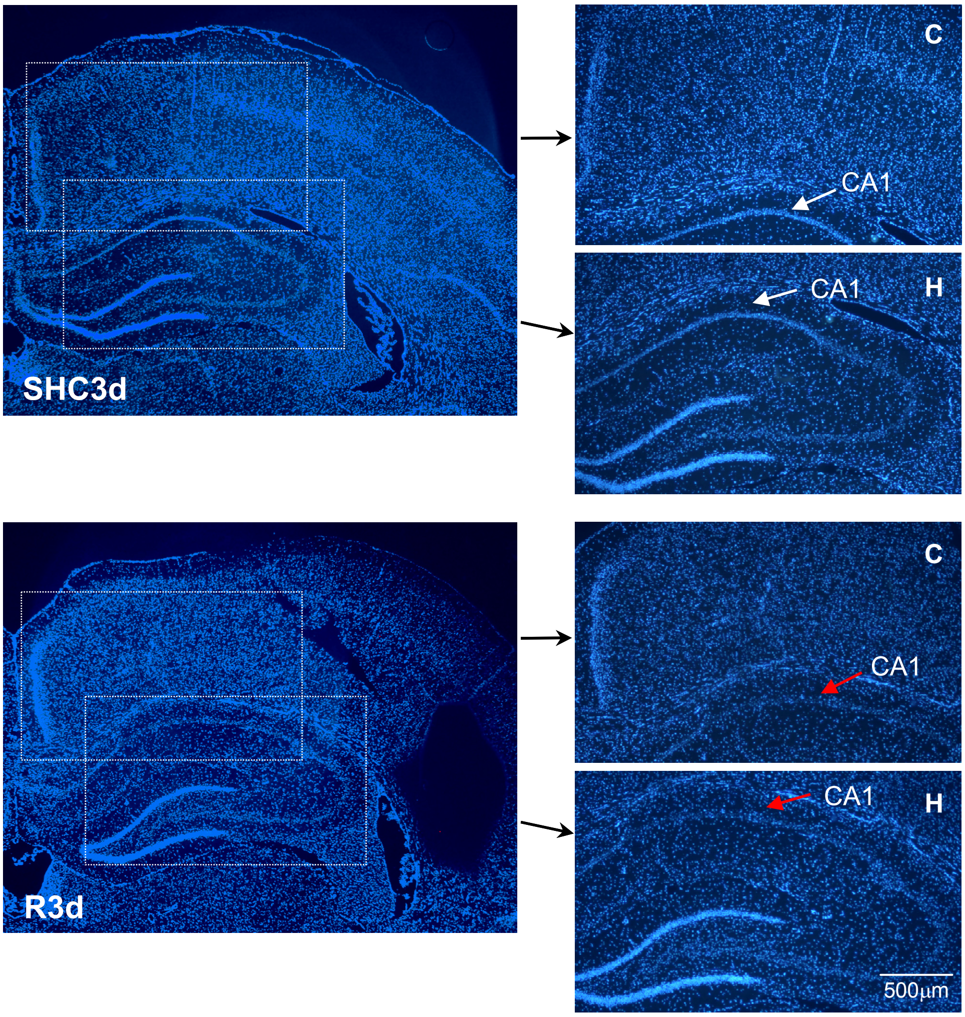

Figure S5. Representative stained brain sections from the animal model of transient cerebral ischemia. Images of stained brain sections from 3-day sham control animals (SHC3d) and ischemic animals with 3 days of reperfusion (R3d) (left images), were magnified to show the cerebral cortex (C) and hippocampal (H) regions (right images). The hippocampal *cornu ammonis* 1 (CA1) region is indicated by arrows. Note the cell damage of the CA1 region induced in R3d ischemic animals (red arrows). Brain sections are from the experiment in Figure 5 and stained with Hoechst 33342.
